# Fluorescence-guided craniotomy of glioblastoma using panitumumab-IRDye800

**DOI:** 10.3171/2021.10.FOCVID21201

**Published:** 2022-01-01

**Authors:** Quan Zhou, Gordon Li

**Affiliations:** Departments of 1 Neurosurgery and; 2 Otolaryngology–Head & Neck Surgery, Stanford University School of Medicine, Stanford, California

**Keywords:** glioblastoma, near-infrared, fluorescence imaging, panitumumab-IRDye800, craniotomy, epidermal growth factor receptor

## Abstract

A contrast-enhancing lesion in the left temporal lobe of a 72-year-old woman was biopsied and diagnosed as glioblastoma. Near-infrared (NIR)–labeled epidermal growth factor receptor (EGFR) antibody, panitumumab-IRDye800, was infused 52 hours before craniotomy without pretreatment. Tumor fluorescence was detected through intact dura, and the visual contrast between disease and peritumoral healthy brain was enhanced after tumor exposure. Residual cancerous tissue was identified with strong fluorescence in resection cavity after en bloc tumor removal. Minimal fluorescence remained in the final wound bed, likely from nonenhancing tumor. Fluorescence was heterogeneously distributed at the infiltrative margin in resected tumor pieces imaged ex vivo. Postoperative MRI confirmed gross-total resection.

The video can be found here: https://stream.cadmore.media/r10.3171/2021.10.FOCVID21201

## Transcript

Fluorescence-guided craniotomy of glioblastoma using panitumumab-IRDye800 by Drs. Quan Zhou and Gordon Li.

### 0:31 Left Temporal Glioblastoma.

Contrast-enhancing glioblastoma in the left temporal lobe of patient was found on preoperative MRI.

### 0:38 Panitumumab-IRDye800 Infusion.

Fifty milligrams of near-infrared labeled EGFR antibody, panitumumab-IRDye800, was infused without preloading of unlabeled antibody 2 days before surgery within the imaging window of 1–5 days.^1–4^

### 0:54 Skull Removed to Expose Dura.

On the day of surgery, patient skull was removed to reveal dura. The tumor is located immediately beneath the intact dura.

### 1:14 SPY-Phi: NIR.

A portable handheld device was used to acquire near-infrared images of the surgical field.^5^

### 1:34 Tumor Exposed: White Light.

Next, the dura was open to expose the tumor under white light illumination.

### 1:44 Intraoperative Neuronavigation.

Intraoperative neuronavigation mapped the location of tumor surface onto stereotactic coordinates of preoperative MRI scan.

### 1:54 Tumor Exposed: NIR.

Exposed tumor was then illuminated with near-infrared light and fluorescence signal was collected with the handheld imager.

### 2:08 Residual Tumor.

Residual tumor was identified with high fluorescence intensity and removed from resection cavity.

### 2:15 Wound Bed.

Minimal fluorescence, likely from nonenhancing tumor, remained in the final wound bed outside the contrast-enhancing margin on MRI, and thus not removed per standard-of-care protocol.

### 2:25 IGP: NIR.

Resected tumor pieces were imaged in a near-infrared instrument free from ambient light.^6^

### 2:35 Postoperative MRI: T1+C.

Postoperative MRI confirmed gross-total resection of glioblastoma.

## Acknowledgments

Institutional equipment loans were received from LI-COR Biosciences. Illustrations were created with BioRender - biorender.com.

This work was supported by NIH R01CA190306 (G.L.).

## Disclosures

Dr. Li reported personal fees from Medtronic, Synthes, and Photonics Medical during the conduct of the study.

## Author Contributions

Primary surgeon: Li. Editing and drafting the video and abstract: Zhou. Critically revising the work: Zhou. Reviewed submitted version of the work: Zhou.
